# Optimization of Gradient-Echo Echo-Planar Imaging for T_2_* Contrast in the Brain at 0.5 T

**DOI:** 10.3390/s23208428

**Published:** 2023-10-12

**Authors:** Arjama Halder, Chad T. Harris, Curtis N. Wiens, Andrea Soddu, Blaine A. Chronik

**Affiliations:** 1 Department of Medical Biophysics, Western University, London, ON N6A 3K7, Canada; 2 Synaptive Medical, Toronto, ON M5V 3B1, Canada; 3 Western Institute for Neuroscience, Physics and Astronomy, Western University, London, ON N6A 3K7, Canada; asoddu@uwo.ca

**Keywords:** mid-field MRI (<1 T), functional MRI, gradient-echo EPI, T_2_* contrast efficiency, SNR, BOLD, multi-echo EPI, gradient system, slew rate

## Abstract

Gradient-recalled echo (GRE) echo-planar imaging (EPI) is an efficient MRI pulse sequence that is commonly used for several enticing applications, including functional MRI (fMRI), susceptibility-weighted imaging (SWI), and proton resonance frequency (PRF) thermometry. These applications are typically not performed in the mid-field (<1 T) as longer T_2_* and lower polarization present significant challenges. However, recent developments of mid-field scanners equipped with high-performance gradient sets offer the possibility to re-evaluate the feasibility of these applications. The paper introduces a metric “T_2_* contrast efficiency” for this evaluation, which minimizes dead time in the EPI sequence while maximizing T_2_* contrast so that the temporal and pseudo signal-to-noise ratios (SNRs) can be attained, which could be used to quantify experimental parameters for future fMRI experiments in the mid-field. To guide the optimization, T_2_* measurements of the cortical gray matter are conducted, focusing on specific regions of interest (ROIs). Temporal and pseudo SNR are calculated with the measured time-series EPI data to observe the echo times at which the maximum T_2_* contrast efficiency is achieved. T_2_* for a specific cortical ROI is reported at 0.5 T. The results suggest the optimized echo time for the EPI protocols is shorter than the effective T_2_* of that region. The effective reduction of dead time prior to the echo train is feasible with an optimized EPI protocol, which will increase the overall scan efficiency for several EPI-based applications at 0.5 T.

## 1. Introduction

Modern mid-field MRI (0.3 < B < 1 T) scanners offer widespread diagnostic use for a range of applications, including the identification of neurological diseases, especially in an acute setting [1,2,3]. In addition to these advantages, specialized mid-field systems for head imaging offer smaller size, lighter weight, and compact fringe field, enabling easier siting and installation in locations close to the vulnerable patient population and offering unique application scenarios, such as point-of-care and intraoperative monitoring [1].

Relaxation parameters vary as a function of magnetic field strength, significantly impacting the optimal acquisition strategy. Relative to typical clinically used field strengths (1.5 T and 3.0 T), relaxation parameters at 0.5 T display a shortened T_1_ relaxation time and prolonged T_2_* relaxation time [3]. For example, the T_1_ of gray matter reduced from 1304 ms at 1.5 T to 717.2 ms at 0.55 T, and the T_2_* of gray matter increased from 68 ms at 1.5 T to 86.4 ms at 0.55 T [4]. Shortened T_1_ relaxation times promote acquisitions with short TRs, while a prolonged T_2_* extends data sampling schemes, such as echo planar, spiral, and multiple echo acquisitions.

Advantageous relaxation properties, short T_1_ and long T_2_* [4], and reduced susceptibility-induced field inhomogeneities at mid-field strength make gradient-recalled echo (GRE) echo-planar imaging (EPI) an enticing sequence for several applications, including susceptibility-weighted imaging [5,6], perfusion [6], functional imaging (fMRI) [7,8], and proton resonance frequency (PRF) thermometry [9]. These advantageous relaxation properties were recently leveraged in diffusion-weighted EPI to help overcome the reduced signal-to-noise ratio inherent to lower-field-strength systems caused by reduced polarization [2]. Generally, low bandwidth in the phase encode direction implicit to EPI makes it prone to susceptibility-induced distortions; however, this effect is significantly reduced in the mid-field. The reduction in susceptibility-induced field inhomogeneities improves image geometric fidelity, particularly in regions near air–tissue interfaces, such as air cavities and the skull base [2].

Functional MRI at the point of care is a tantalizing tool since it can provide vital neurological information in cases of traumatic brain injury [10] and acute ischemic stroke [11]. This technique is typically reserved for higher-field-strength (>1.5 T) systems due to the increase in magnetic susceptibility contrast and signal-to-noise ratio (SNR) with field strength [12]. Despite this, many applications of fMRI are possible with modern, high-performance mid-field systems. For example, recent studies demonstrated the feasibility of motor-task-based fMRI [8] and visual-task-based fMRI [7] at 0.5 T and 0.55 T, respectively.

The purpose of this work was to optimize GRE EPI in the brain at 0.5 T with a metric that minimizes dead time in the sequence while maximizing T_2_* contrast. This optimization guides the sequence parameters to be chosen for future task-based and resting-state fMRI and thermometry studies. T_2_* measurements of overall gray matter (GM) and white matter (WM) were quantified, and theoretical protocol optimizations were performed to maximize T_2_* contrast efficiency. To validate the theoretical protocol optimizations and identify the contributions of physiological noise, temporal and pseudo SNR measurements for 3.4 mm and 4.0 mm isotropic echo-planar imaging acquisitions at multiple echo times were recorded.

## 2. Theory

For functional MRI, the SNR related to GRE EPI sequences, including the BOLD contribution, has been defined previously [13] as:

(1) SNRBOLD∝∆x∆y∆zTETAD(1−e−TR/T1)sin⁡(α)1−cos⁡(α)e−TR/T1e−TET2*

where ∆x, ∆y, and ∆z are the dimensions of the imaging voxel, $T_{AD}$ is the duration of the acquisition readout, α is the flip angle, TR is the repetition time, TE is the echo time, and $T_{1}$ and $T_{2}^{*}$ are the longitudinal and transverse relaxation times, respectively.

During an fMRI experiment, multiple images are acquired in a time series within a total scan time, and *N* here is the total number of images. Including the averaging effect of the time series and assuming uncorrelated noise across images, the BOLD SNR becomes [13]:

(2) SNR~BOLD∝∆x∆y∆zTENTAD(1−e−TR/T1)sin⁡(α)1−cos⁡(α)e−TR/T1e−TET2*=NSNRBOLD

For a fixed scan duration with a total scan time TS = N*TR, Equation (2) can be re-written as:

(3) $${\tilde{SNR}}_{BOLD}=\sqrt{\frac{TS}{TR}}{SNR}_{BOLD}=\sqrt{TS}\cdot \eta_{T_{2}^{*}}$$

where $\eta_{T_{2}^{*}}$ is the efficiency of T_2_* contrast, given by:

(4) $$\eta_{T_{2}^{*}}=\frac{{SNR}_{BOLD}}{\sqrt{TR}}$$

$\eta_{T_{2}^{*}}$ can be used to compare pulse sequence timing parameters for a given T_2_* value, where optimal parameters are found when $\eta_{T_{2}^{*}}$ is maximized.

## 3. Methods

All imaging protocols were performed on a head-only 0.5 T MR scanner equipped with a high-performance gradient system and 16-channel head coil (Synaptive Medical, Toronto, ON, Canada). Imaging was performed on five healthy volunteers (male, age = 38 ± 8) with informed consent in compliance with health and safety protocols. For all measurements, subjects were asked to relax while in the scanner with their eyes closed.

### 3.1. Segmentation of 3D T_1_-Weighted Imaging

Structural images were acquired at 1.1 mm isotropic resolution using a T_1_-weighted MPRAGE sequence with field of view (FOV) = 236 mm × 236 mm × 180 mm, zipped to a matrix size = 512 × 512 × 320, flip angle (FA) = 9°, T_E_ = 5 ms, and T_R_ = 11.2 ms.

Region of interest (ROI) masks were created using anatomical T_1_-weighted images. First, the Brain Extraction Tool (BET) [14] from FMRIB Software Library (FSL) was applied to obtain the skull-stripped brain. Manual adjustments were performed to maximize cortical coverage on the skull-stripped brain, which was then run through FMRIB’s Automated Segmentation Tool (FAST) [15] in FSL to create cortical GM and WM masks.

A visual cortex mask was created using the Juelich histological atlas [16,17,18,19,20] in Montreal Neurological Institute (MNI) 152 standard space (resolution = 2 mm). These masks were then thresholded at an intensity of 30 and nonlinearly registered onto the anatomical image using FMRIB’s Nonlinear Image Registration Tool (FNIRT) [21].

### 3.2. T_2_* Mapping of Segmented Regions

A 2 mm isotropic resolution 3D multi-echo GRE (meGRE) sequence was used with FOV = 240 mm × 240 mm × 120 mm, matrix size = 120 × 120 × 60, FA = 32°, T_E_ (1) = 5 ms, echo spacing = 3.4 ms, echo train length (ETL) = 26, and T_R_ = 97.25 ms.

The 3D meGRE images were registered to the anatomical image using FMRIB’s Linear Image Registration Tool (FLIRT) [22,23]. Voxel-wise estimates of T_2_* were computed over the ROIs by fitting the data to a mono-exponential decay model [24].

### 3.3. EPI of Segmented Regions

Time-series EPI data were collected at two isotropic spatial resolutions (3.4 mm and 4 mm) for nine different protocols for five healthy volunteers with varying echo times (range = 25–105 ms, with an interval of 10 ms). The following parameters were constant for all EPI scans: in-plane FOV = 240 mm × 240 mm, and acquisition bandwidth = 160 kHz. Parameters specific to the 3.4 mm protocols were: ETL = 70; number of slices = 38; slice FOV = 129.2 mm; FA = [85, 87, 88, 89, 89, 90, 90, 90, 90] degrees; T_R_ = [1738, 2119, 2499, 2879, 3259, 3639, 4019, 4399, 4779] ms. Similarly, parameters specific to the 4.0 mm protocol were: ETL = 60; number of slices = 32; slice FOV = 128 mm; FA = [82, 85, 87, 88, 89, 89, 89, 90, 90] degrees; T_R_ = [1386, 1706, 2026, 2346, 2666, 2986, 3306, 3626, 3946] ms. Flip angles were chosen to be the Ernst angle for the given acquisition T_R_ for GM at 0.5 T [4].

For each acquisition, temporal SNR (tSNR) and pseudo multiple replica SNR (pSNR) [25] were computed after linear registration to the anatomical image. tSNR was computed by performing 64 repeat acquisitions, correcting for motion using MCFLIRT [24], and computing their variance voxel-wise through the time series. Conversely, pSNR computed a pseudo time series from a single acquisition and the noise correlation matrix. Prior to reconstruction, 128 repetitions of properly scaled and correlated noise were added to k-space data to create a pseudo time series. Images in this time series only differ due to thermal noise and do not contain any effects due to physiological noise or system instability.

The mean and standard deviation of the tSNR and pSNR values for each ROI were calculated. These values were converted to $\eta_{T_{2}^{*}}$ and compared to theoretical values computed using Equation (4). Also, these values were used to compute the ratio of physiological noise to thermal noise [26], as defined by Equation (5) below, to understand the noise regime of the acquisitions.

(5) $$\frac{\sigma_{P}}{\sigma_{T}}=\sqrt{{\left(\frac{pSNR}{tSNR}\right)}^{2}-1}$$

## 4. Results

**Figure 1:**
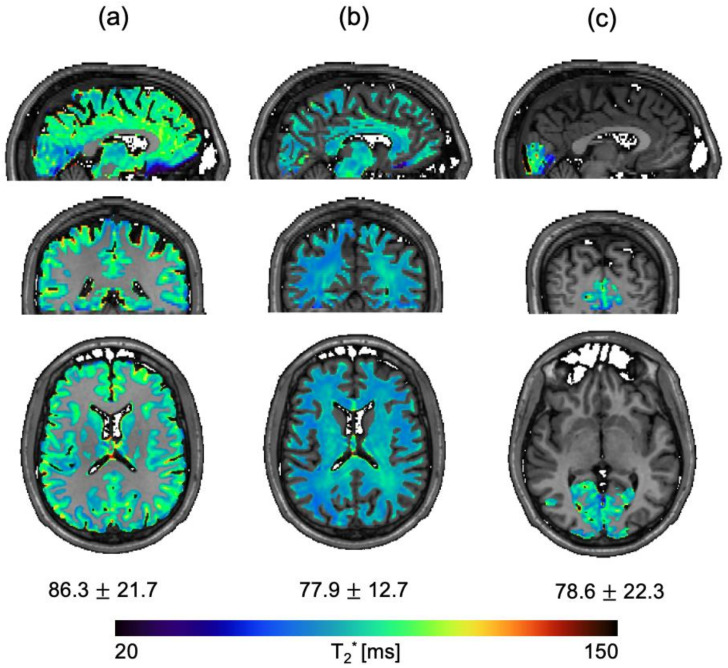
T_2_* maps overlaid on a T_1_-weighted MPRAGE of a healthy volunteer are shown in sagittal, coronal, and axial planes for the following regions: gray matter (**a**), white matter (**b**), and gray matter overlapped with the visual cortex (**c**). The cumulative mean and standard deviation T_2_* measurement acquired with five healthy volunteers are shown at the bottom of each column.

Figure 1 shows an example segmented T_2_* map overlaid on top of a T_1_-weighted anatomical image of one of the volunteers. For each segmented region, sagittal, coronal, and axial formats are shown. The cumulative mean and standard deviation calculated with the five volunteers are presented at the bottom of each column.

**Figure 2:**
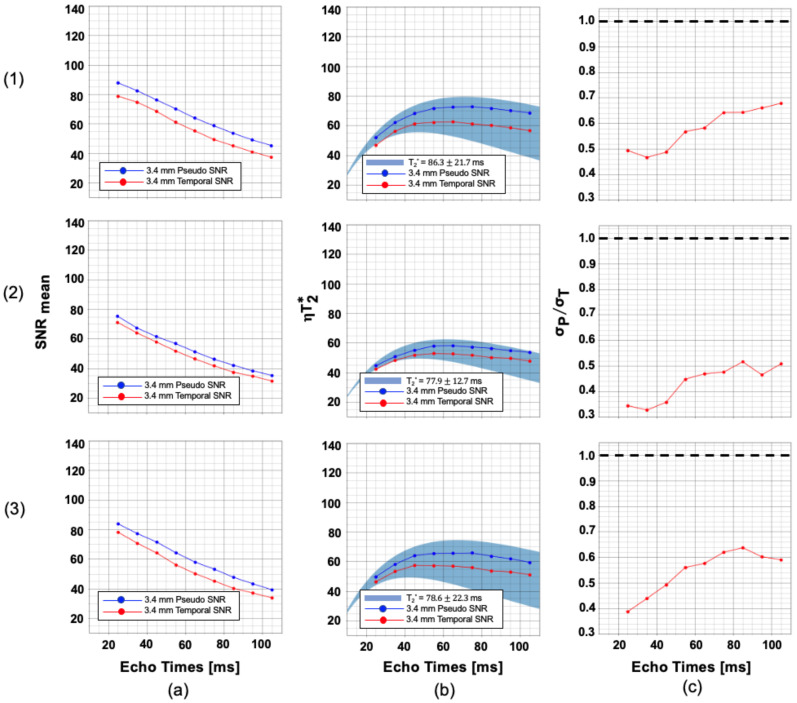
Sequence optimization plots for 3.4 mm isotropic resolution. (**a**) Mean temporal SNR and pseudo-replica SNR; (**b**) T_2_* efficiency; and (**c**) physiological-to-thermal noise ratio plotted against echo time for rows: (**1**) gray matter; (**2**) white matter, and (**3**) gray matter overlapped with the visual cortex. The dashed line in (c) shows the threshold $\sigma_{P}/\sigma_{T}$ < 1 at all times signifies thermal noise dominance.

For all EPI scans, the highest absolute and relative mean displacements due to motion were found to be less than 0.2 mm. Figure 2 shows the pseudo-replica (blue) and temporal SNR (red), T_2_* efficiency, and physiological to thermal noise variation as a function of T_E_ averaged over five volunteers for a 3.4 mm isotropic protocol. Pseudo-replica SNR, which contains only thermal noise, demonstrates a reduction in SNR as echo times increase in all ROIs. Furthermore, the temporal SNR, which contains contributions from both thermal and physiological noises, is consistently lower than the pseudo-replica SNR. These curves do not follow a simple exponential curve, as increasing the T_E_ necessitates an increase in T_R_ and FA as well. Measured T_2_* efficiencies, derived from the pSNR, and theoretical T_2_* efficiencies (Equation (4)) demonstrate good agreement. Physiological-to-thermal noise ratios are relatively small and increase as T_E_ approaches T_2_*; however, thermal noise dominates for all echo times measured.

**Figure 3:**
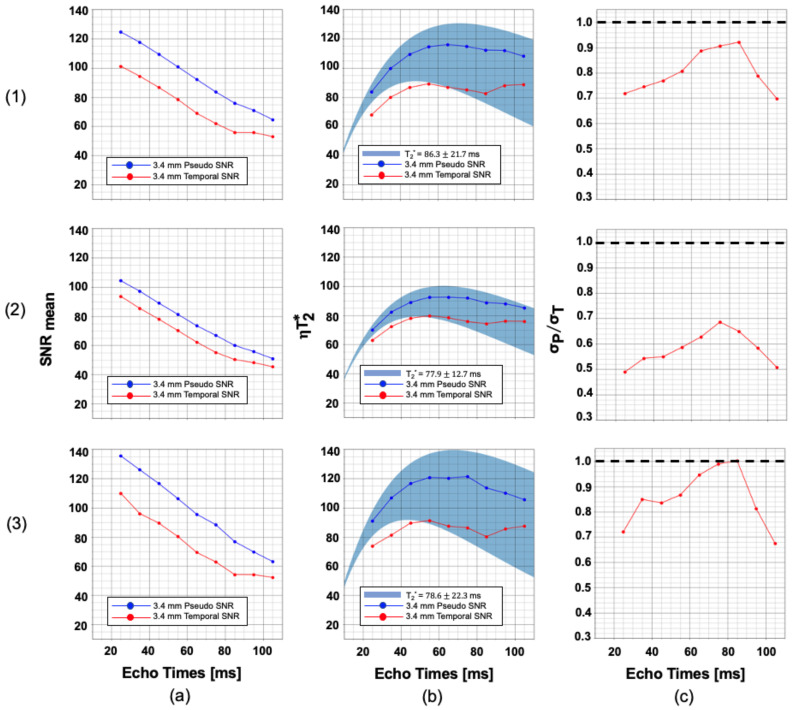
Sequence optimization plots for 4.0 mm isotropic resolution. (**a**) Mean temporal SNR and pseudo-replica SNR; (**b**) T_2_* efficiency; and (**c**) the physiological-to-thermal noise ratio plotted against echo time for rows: (**1**) gray matter, (**2**) white matter, and (**3**) gray matter overlapped with the visual cortex. The dashed line in (c) shows the threshold $\sigma_{P}/\sigma_{T}$ < 1 at all times signifies thermal noise dominance.

Figure 3 shows the pseudo-replica (blue) and temporal SNR (red), T_2_* efficiency, and physiological to thermal noise variation as a function of T_E_ averaged over five volunteers for a 4.0 mm isotropic protocol. For this protocol, a substantial drop is observed when comparing pseudo-replica and temporal SNR. This suggests that physiological noise in this protocol is now a substantial contributor. This observation can also be seen in the significant deviation of the tSNR efficiency curve from the theoretical curve and the high physiological-to-thermal noise ratio.

As expected, tSNR and derived efficiency curves deviate significantly more from their pSNR counterparts at 4 mm resolution than at 3.4 mm resolution. This is due to the increase in physiological noise relative to thermal noise as the voxel size and baseline SNR increase. As can be seen in column (c) of Figure 3, this effect is particularly evident for echo times close to T_2_*, in agreement with prior work [26]. Thermal noise dominates over physiological noise for all measurements except when T_E_ ≈ T_2_* in the visual cortex regions.

## 5. Discussion

The T_2_* relaxation parameter was measured at 2 mm isotropic resolution in the following regions: gray matter, white matter, and visual cortex. The measurements of gray (86.3 ± 21.7 ms) and white matter (77.9 ± 12.7 ms) are in good agreement with recent measurements of gray (86 ± 9 ms) and white matter (72 ± 12 ms) acquired at 0.55 T [4]. To our knowledge, an estimate of the T_2_* relaxation in the visual cortex (78.5 ± 22.3 ms), specifically at 0.5 T, is not recorded elsewhere in the literature.

According to theory, optimal T_2_* contrast occurs at TE = T_2_*, which has resulted in long echo times for previously reported fMRI validation studies in the mid-field [7,8]. However, the results from this work indicate that for single-echo GRE EPI at 0.5 T, the optimal echo time for T_2_* contrast efficiency occurs at TE < T_2_* for practical readout bandwidths and slice coverage. This is because the T_2_* is so long in the mid-field that dead time can occur between excitation and acquisition when echo train lengths are short (as is the case with the modest resolution for fMRI). For example, for gray matter, the 3.4 mm and 4 mm protocols investigated in this work have a T_2_* efficiency-optimal echo time of 55 ms, a reduction of 31 ms when compared to a T_2_* contrast-optimal TE (i.e., TE = T_2_* = 86 ms). This results in a significant reduction in T_R_ of around 600 ms.

Equation (4) shows that the T_2_* contrast efficiency scales with 1/$\sqrt{T_{R}}$, which suggests higher efficiency with reduced T_R_. These shorter repetition times are also feasible with this 0.5 T scanner since T_1_ is shorter. In this work, the flip angle changes moderately due to the coverages used in these experiments, so the deviation measured in the signal-to-noise ratio from the mono-exponential decay is dominated by the change in T_R_.

In this work, protocol optimization was achieved in part by minimization of sequence dead time. It is important to note that dead time is not always an undesired feature of a pulse sequence. Dead time results in reduced SAR and duty cycle of the RF or gradient amplifiers; however, these problems are not an issue for the head-only 0.5 T system used in this work. SAR is a factor of nine times less than at 1.5 T, and the RF transmit and gradient coils of this system lie in close proximity to the subject, providing greater efficiency and hence reduced hardware limitations. Therefore, in the mid-field (and, in particular, when using a head-only scanner equipped with high-performance gradient coils), the reduction of dead time is directly related to improving scanner efficiency.

By simply reducing the readout bandwidth, long echo times with minimal sequence dead time can be achieved. However, this comes at the cost of increased image distortions [27]. This can be illustrated by way of example. Consider the case where T_2_* = 90 ms and ETL = 60 (4 mm resolution); to achieve T_E_ = T_2_* without sequence dead time, the echo spacing between phase encode lines would need to be approximately 3 ms, corresponding to a phase encode bandwidth of 5 Hz/pixel. In comparison, the 4 mm protocol used in this work had a phase encode bandwidth of ~43 Hz/pixel, a factor of 8.6 improvement in geometric distortion.

Alternatively, instead of applying a lower bandwidth, one could use a multi-echo readout to eliminate sequence dead time. Multi-echo GRE EPI (meGRE EPI) [28,29,30,31] increases the acquisition duty cycle, while maintaining long T_E_ and reduced geometric distortion. Furthermore, since meGRE EPI benefits from a high slew rate [27,32,33], it is an ideal sequence modification for a head-only system equipped with high-performance gradients, as was the system used in this study [34]. A detailed comparison of T_2_* contrast efficiency between single-echo and multi-echo EPI at 0.5 T will be presented in a future publication.

The average pSNR and tSNR from five volunteers were used to compute the physiological-to-thermal noise ratio ($\sigma_{P}/\sigma_{T}$) for two resolutions across multiple echo times. At 3.4 mm resolution, thermal noise dominance was observed for all echo times measured over all regions of interest. At 4 mm resolution, very slight physiological noise dominance was observed in the visual cortex at echo times close to the region’s measured T_2_* value. These results suggest that any improvement in SNR from high-performance RF coil design or multi-echo EPI protocols will translate into significant improvement in tSNR at voxel sizes ≤ 64 mm^3^. Furthermore, only modest tSNR improvement can be gained by increasing the voxel size further; however, this would result in greater partial volume corruption, potentially negating any benefits from increased tSNR.

These results allow the quantification of the T_2_* contrast efficiency, which is essentially a metric that can be used to optimize parameters of the GRE EPI sequence for fMRI application at 0.5 T. The optimized parameters can then be used for task-based or resting-state experiments in the future. A similar analysis was performed for PRF thermometry applications in [9], which also showed peak-temperature precision efficiency values at T_E_, lower than T_2_*. This optimized protocol can be used for other EPI-based applications such as SWI [5].

## 6. Conclusions

For peak T_2_* contrast efficiency, the optimal echo time for GRE EPI sequences implemented at 0.5 T will be less than T_2_* when echo train lengths are short and dead time would otherwise be present in the sequence. For further improvement of GRE EPI at 0.5 T, multi-echo EPI is an enticing option as it will reduce sequence dead time while maintaining the long echo times necessary for T_2_* contrast.

## Data Availability

The data presented in this study are available on request from the corresponding author. The data are not publicly available due to confidentiality reasons.
